# SQSTM1 Mutations and Glaucoma

**DOI:** 10.1371/journal.pone.0156001

**Published:** 2016-06-08

**Authors:** Todd E. Scheetz, Ben R. Roos, Frances Solivan-Timpe, Kathy Miller, Adam P. DeLuca, Edwin M. Stone, Young H. Kwon, Wallace L. M. Alward, Kai Wang, John H. Fingert

**Affiliations:** 1 Department of Ophthalmology and Visual Sciences, Carver College of Medicine, University of Iowa, Iowa City, Iowa, United States of America; 2 Stephen A. Wynn Institute for Vision Research, University of Iowa, Iowa City, Iowa, United States of America; 3 Department of Biostatistics, College of Public Health, University of Iowa, Iowa City, Iowa, United States of America; Purdue University, UNITED STATES

## Abstract

Glaucoma is the most common cause of irreversible blindness worldwide. One subset of glaucoma, normal tension glaucoma (NTG) occurs in the absence of high intraocular pressure. Mutations in two genes, optineurin (*OPTN*) and TANK binding kinase 1 (*TBK1*), cause familial NTG and have known roles in the catabolic cellular process autophagy. TKB1 encodes a kinase that phosphorylates OPTN, an autophagy receptor, which ultimately activates autophagy. The sequestosome (*SQSTM1*) gene also encodes an autophagy receptor and also is a target of TBK1 phosphorylation. Consequently, we hypothesized that mutations in *SQSTM1* may also cause NTG. We tested this hypothesis by searching for glaucoma-causing mutations in a cohort of NTG patients (n = 308) and matched controls (n = 157) using Sanger sequencing. An additional 1098 population control samples were also analyzed using whole exome sequencing. A total of 17 non-synonymous mutations were detected which were not significantly skewed between cases and controls when analyzed separately, or as a group (p > 0.05). These data suggest that *SQSTM1* mutations are not a common cause of NTG.

## Introduction

Glaucoma is an important public health problem that is the common cause of irreversible blindness worldwide [1]. Glaucoma is a disease of the optic nerve, which carries visual signals from the retina in the eye to the brain. The key features of glaucoma are damage to the optic nerve, which is visible on ophthalmic exam, and a characteristic pattern of visual field loss. The most common type of glaucoma in many nations is primary open angle glaucoma (POAG), which occurs in the absence of any other ocular abnormalities including anatomical defects that might obstruct fluid egress from the eye. High intraocular pressure (IOP) is a potent risk factor for glaucoma [2]. The higher the IOP, the greater the risk for developing glaucoma, however, glaucoma can occur at any IOP [3]. When glaucoma occurs below an arbitrary threshold pressure, 21 mm Hg, it is frequently termed normal tension glaucoma (NTG).

The basic causes of NTG have been explored with genetic studies. Population-based studies using genome-wide association studies have identified several risk factors that contribute to the development of complex genetic forms of NTG, including S1 RNA binding domain 1 (*SRBD1*) [4], elongation of long chain fatty acids family member 5 (*ELOVL5*) [4], toll-like receptor 4 (*TLR4*) [5], and *CDKN2B-AS1* [6,7]. These NTG risk factors are present in NTG patients at a statistically higher frequency than they are observed in individuals with healthy eyes [8].

Genetic studies have also been conducted on large families with numerous members diagnosed with NTG. Linkage analysis of one such pedigree identified optineurin (*OPTN*) as an NTG-causing gene. One mutation in *OPTN*, E50K, was subsequently associated with 1–2% of cases of NTG [9–11]. More recently, study of another large NTG pedigree showed that copy number variation (duplication) of the TANK binding kinase 1 (*TBK1*) gene is another cause of NTG [12]. Population studies later demonstrated that copy number variations in *TBK1* (duplications, triplications, and one deletion) are associated with approximately 1% of NTG cases [12–16]. The E50K *OPTN* mutation and *TBK1* copy number variations are high penetrance mutations. Individuals with either an *OPTN* mutation or a *TBK1* mutation almost always develop NTG, while these mutations are only rarely observed in normal individuals [8].

### NTG genes and autophagy

Both NTG genes (*TBK1* and *OPTN*) have important roles in autophagy suggesting that macroautophagy may be a key mechanism in NTG. Macroautophagy, hereafter referred to as autophagy, is an ancient catabolic cellular process that digests intracellular structures for energy in times of nutritional deprivation [17]. The same mechanisms are also used by cells to eliminate accumulating proteins or intracellular pathogens [17–19]. In autophagy a double membrane endosome, the autophagosome, forms around targeted materials within the cytoplasm. The autophagosome fuses with the lysosome and the contents of the resulting autophagolysosome are degraded. Decreased levels of autophagy have been associated with neurodegenerative diseases including Parkinson disease, Huntington disease, and amyotrophic lateral sclerosis (ALS) with the presumption that reduced ability to eliminate accumulating materials in neurons leads to their death and disease [20–24]. Alternatively, increased autophagy has been observed in animal models of glaucoma and optic nerve disease [25,26]. In this case, excess catabolic activity from autophagy may destroy cellular components necessary for survival [25]. Consequently, defects in *TBK1* or *OPTN* and autophagy are a plausible cause of the retinal ganglion cell death and optic nerve damage that are central features of glaucoma.

*TBK1* encodes a kinase that stimulates autophagy by phosphorylating OPTN at Ser177 [18]. Phosphorylation of OPTN activates its functional domains to link structures targeted for elimination with proteins of an assembling autophagosome. Specifically OPTN’s ubiquitin binding domain associates with ubiquitinated targets of autophagy and its LC3 binding domain associates with a key component of the forming autophagosome, i.e. microtubule-associated protein light chain 3 (LC3). In this way OPTN acts as an autophagy receptor, linking targets of autophagy with specific elements of the autophagosome and promotes autophagy [18].

There is evidence that NTG-causing mutations in *OPTN* and *TBK1* promote increased activation of autophagy. Yeast two-hybrid studies indicate that an NTG-causing *OPTN* mutation, enhances the association between OPTN and TBK1 [27], which would likely increase the opportunity for TBK1 to phosphorylate OPTN and stimulate autophagy. Also, all but one [15] of the *TBK1* CNVs discovered in NTG patients to date are duplications and triplications, which would similarly favor activation of OPTN and autophagy. Finally, we have studied autophagy in retinal ganglion cell-like neurons that were generated using skin biopsies collected from patients with NTG caused by a *TBK1* duplication using induced pluripotent stem cell methods. Key markers of autophagy are increased in retinal ganglion cell-like neurons with *TBK1* gene duplications [28].

The discovery that two autophagy genes cause NTG suggests that additional autophagy genes may also be important in glaucoma. One autophagy gene, sequestosome 1 (*SQSTM1*) or p62, is an especially good candidate for causing NTG. There are similarities with *OPTN* that suggest *SQSTM1* may also be an NTG gene. Both *OPTN* and *SQSTM1* encode proteins that are phosphorylated by TBK1 and function as autophagy receptors [18,29]. Moreover, the genes that cause ALS overlap with the genes that cause NTG. Previous studies have shown that mutations in *TBK1* [30,31], *OPTN* [32] and *SQSTM1* [33,34] are associated with ALS, suggesting a general link between the genetics of ALS and NTG. These data suggest that while some *SQSTM1* mutations are associated with ALS, other mutations may cause NTG. Consequently, we investigated *SQSTM1* for glaucoma-causing mutations in a cohort of NTG patients.

## Materials and Methods

### Patient Cohorts

Subjects enrolled in the study provided written informed consent and the research was approved by the University of Iowa’s Internal Review Board. A cohort of 308 patients with NTG had excavation of their optic nerve head with resultant glaucomatous visual field loss in at least one eye. Glaucomatous optic nerves had cup-to-disc ratios of greater than 0.7 with thinning of the neural rim, asymmetry of the optic nerve cup-to- disc ratio greater than 0.2, or progressive loss of the neural rim identified with baseline disc photography. Patients were 40 years of age or older at diagnosis and had gonioscopically open iridocorneal angles (angle greater than Shaffer grade II). Enrollment criteria included a maximum IOP of 21mmHg or less. A cohort of 157 control subjects over 50 years of age were examined by board-certified ophthalmologists and were judged to have normal optic nerve head appearance and of IOP 21 mm Hg or less. An additional cohort of 1098 patients enrolled in an inherited eye disease study at the University of Iowa were used as a population control cohort. These patients have ocular diagnoses unrelated to glaucoma (90% have photoreceptor degenerations or macular dystrophies).

### DNA sequencing

The entire coding sequence of *SQSTM1* was PCR amplified from DNA samples collected from NTG patients (n = 308) and normal control subjects (n = 157) using overlapping primer pairs in standard PCR reactions (primers listed in Table 1). Amplified DNA sequences were determined using BigDye chemistry and a 3730 automated sequencer (Applied Biosciences, Foster City, CA) as previously described [12]. The coding sequence of *SQSTM1* was determined from DNA samples collected from a cohort of patients with retinal degenerations (n = 1098) using whole exome sequencing with the Agilent v5 kit (Santa Clara, CA) and the Illumina HiSeq2000/2500 (San Diego, CA) and subjected to quality control metrics as previously described [35]. The reads were aligned to the genome using BWA [36], and resulting variants were called using the genome analysis toolkit (GATK) [37]. Variants were annotated and prioritized for follow-up using methods we have described previously [38]. Briefly, variants were first filtered to retain those predicted to alter coding sequence or impact a splice site based upon the RefSeq transcript (NM_003900.4). All variants detected in the *SQSTM1* gene by exome sequencing of this Iowa Population Control cohort are listed in S1 Table. Variants that were seen in at least 1% in any ExAC population [39] were assumed to be too common to cause disease and were removed.

**Table 1:** SQSTM1 primer sequences.

	Forward Primer	Reverse Primer
Exon 1	GGAAGGGGAGAGTAGTGAAGG	CTTGGTCACCACTCCAGTCA
Exon 2	AGCCCTGTGAGTGTCCCTTT	ACAGCCCTCAAATTGCTGAC
Exon 3 / 4	GCAGTGACAGCCCCACAGT	GGCTGCCTGACTACTGTCAC
Exon 5	GACCTTGGCAAGAAGGTGAC	CAGTATTCCAGGTGAAAGTTACATAAA
Exon 6	CTTGCAGGTGCATCCTTGG	GTGCAGGCCACAGATCACTA
Exon 7	CCTAGACCCCTGCAGCCTTA	AGGGCAGGATGCTCTAAAGG
Exon 8	GGGTATGTGTTTCGGGTCAC	TCCTGGAAGAAGGCAGAGAA

These primer sequences were used both for PCR amplification and for Sanger sequencing of the *SQSTM1* coding sequences.

### Statistics

Power calculations were conducted to assess our ability to detect *SQSTM1* mutations. Given the cohort sizes, we had 80% power to detect a statistically significant skew in non-synonymous variants if they occurred at a frequency of 3.5% or greater in the NTG population (n = 308) than in the control population (n = 157). Similarly, this study had 80% power to detect a statistically significant skew in non-synonymous variations if they occurred at a frequency 1.2% or greater in the NTG population (n = 308) than in the population control group (n = 1098). The frequency of non-synonymous variants were compared using Fisher’s exact test with a threshold of 0.05 for significance.

## Results

A cohort of 308 NTG patients and two control cohorts (157 normal controls and 1098 population controls) were tested for glaucoma-causing mutations in the coding sequences of the *SQSTM1* gene using automated Sanger DNA sequencing. Demographic features of these patients are shown in Table 2. A total of 27 unique sequence variations were detected and all were in Hardy-Weinberg equilibrium. Of these variations, 17 were non-synonymous coding sequence mutations (Table 3) and 10 were synonymous changes (Table 4). Two rare non-synonymous mutations (Pro29Ser and Arg212Cys) were detected in the NTG cohort but were absent from both control cohorts. Similarly, eight rare non-synonymous variants were detected only in the control cohorts. A single conservative variant, Glu274Asp, was detected too frequently in NTG patients (4.9%) and in control subjects (6.4% in normal controls and 4.1% in population controls) to be a cause of glaucoma and was excluded from analysis. Non-synonymous variants in *SQSTM1* were detected at a higher frequency in the NTG cohort (3.6%) than in the control cohorts (1.3% in normal controls and 2.9% in population controls). The frequency of *SQSTM1* mutations was 2 to 3-fold higher in NTG patients than in Iowa normal controls, however, this difference in frequency was not statistically significant (p = 0.23). Finally, no statistically significant difference in the frequency of non-synonymous *SQSTM1* mutations was detected when all three cohorts (Iowa NTG, Iowa normal controls, and Iowa population controls) were compared (p = 0.38)

**Table 2:** Patient and control cohort demographics.

	Iowa NTG Cohort	Iowa Normal Controls	Iowa Population Controls
Total	308	157	1098
Female	213	84	589
Male	94	73	507
XXY	0	0	2
Unknown	1	0	0
Mean age at enrollment	69.4	70.1	NA
Diagnosis			
NTG	308	0	NA
Photoreceptor degeneration	NA	NA	834
Maculopathy	NA	NA	148
Congenital cataract	NA	NA	77
Other	NA	NA	39

Abbreviations: not available (NA).

**Table 3:** Non-synonymous *SQSTM1* mutations.

	Iowa NTG Cohort		Iowa Normal Controls		Iowa Population Controls			ExAC European			
	n = 308		n = 157		n = 1098			Non-Finnish	Mutation Analysis		
		Genotype		Genotype		Genotype		Genotype			
Non-synonymous variants	Instances	frequency	Instances	frequency	Instances	frequency	P-value	frequency	SIFT	Blosum62	PolyPhen-2
Pro29Ser	1	0.32%	0	0	0	0	0.30	0.018%	0.07 (tolerated)	-1	0.074 (benign)
Ala33Val	1	0.32%	0	0	2	0.18%	0.65	0.34%	0.23 (tolerated)	0	0.008 (benign)
*c.117_118insG (frameshift)	0	0	0	0	3	0.27%	>0.99	0	N/A	N/A	N/A
Ala117Val	2	0.65%	0	0	4	0.36%	0.65	0.420%	0.34 (tolerated)	0	0.000 (benign)
Pro118Ser	1	0.32%	0	0	1	0.09%	0.51	0.052%	0.54 (tolerated)	-1	0.0090 (benign)
Asn125Ser	0	0	0	0	1	0.09%	>0.99	0	0.29 (tolerated)	1	0.086 (benign)
Val153Ile	1	0.32%	0	0	1	0.09%	0.51	0.066%	0.11 (tolerated)	3	0.011 (benign)
*Arg183Cys	0	0	0	0	1	0.09%	>0.99	0	0.03 (damaging)	-3	0.785 (possibly damaging)
*Arg212Cys	1	0.32%	0	0	0	0	0.30	0	0.09 (tolerated)	-3	0.900 (possibly damaging)
Arg212His	0	0	1	0.64%	0	0	0.10	0.0031%	0.12 (tolerated)	0	0.592 (possibly damaging)
Arg217His	0	0	1	0.64%	0	0	0.10	0.0031%	0.18 (tolerated)	0	0.003 (benign)
*Lys238Glu	3	0.97%	0	0	8	0.73%	0.62	0.54%	0.04 (damaging)	1	0.717 (possibly damaging)
Arg267His	0	0	0	0	2	0.18%	>0.99	0.0060%	0.02 (damaging)	0	0.013 (benign)
Ser275Asn	0	0	0	0	1	0.09%	>0.99	0.27%	0.46 (tolerated)	1	0.716 (possibly damaging)
Ala308Val	0	0	0	0	6	0.55%	0.51	0.0017%	0.11 (tolerated)	0	0.007 (benign)
*Pro392Leu	1	0.32%	0	0%	2	0.18%	0.65	0.26%	0.00 (damaging)	-3	0.988 (probably damaging)
Totals	11	3.6%	2	1.3%	32	2.9%	0.38				
Excluded variants											
Glu274Asp	15	4.9%	10	6.4%	45	4.1%	0.36	4.8%	0.61 (tolerated)	2	0.000 (benign)

Seventeen non-synonymous or frameshift *SQSTM1* mutations were detected in NTG patients, normal control subjects, and population control subjects. Mutations judged likely to be deleterious by causing a truncated protein or as determined by two of the three mutation analysis algorithms (SIFT, blosum62, or PolyPhen) are indicated with asterisks. One mutation at the bottom of the table, Glu274Asp, occurred too frequently in control populations (>1%) and was excluded from analyses. The frequency of each mutation in the non-Finnish Caucasian cohort of the ExAC public database is also reported (exac.broadinstitute.org). All variants in this table have been previously reported in the ExAC database. The c.117_118insC frameshift variant was detected only in the Iowa population control cohort. However, overall, it is not statistically more frequent in this cohort than in the other cohorts (p>0.99), nor did the three individuals with the c.117_188insC frameshift have any known ocular abnormalities in common.

**Table 4:** Synonymous *SQSTM1* mutations.

	Iowa NTG cohort		Iowa Normal Controls		Iowa Population Controls		ExAC European
	n = 308		n = 157		n = 1098		Non-Finnish cohort
Synonymous Variants	Instances	Allele Frequency	Instances	Allele Frequency	Instances	Allele Frequency	Allele Frequency
Gly61Gly	1	0.16%	0	0%	9*	0.41%	-
Ser180Ser	1	0.16%	0	0%	0	0%	0.0092%
Asp292Asp	327	53.1%	161	51%	1185	53.90%	62%
Gly302Gly	0	0%	1	0.32%	4	0.18%	0.026%
Ala308Ala	0	0%	1	0.32%	2	0.09%	0.069%
Arg312Arg	327	53.1%	162	52%	1144	52%	56%
Ser318Ser	16	2.6%	9	2.9%	56	2.50%	3.1%
Ser328Ser	2	0.32%	2	0.64%	10	0.45%	0.55%
Pro348Pro	1	0.16%	1	0.32%	19	0.86%	0.040%
Pro392Pro	1	0.16%	0	0%	1	0.05%	0.0075%

Ten synonymous *SQSTM1* mutations were detected in NTG patients, normal control subjects, and population control subjects. The frequency of each mutation in the non-Finnish Caucasian cohort of the ExAC public database is also reported. All variants in this table have been previously reported in the ExAC database

* DNA sequencing results for this variant were reported for 1092 of 1098 of the Iowa Population.

We also analyzed the predicted deleterious effects of the 17 mutations detected in the *SQSTM1* gene using three algorithms, SIFT, blosum62, and PolyPhen (Table 3). Twelve of the mutations were judged to be benign by at least two of the three algorithms, while four missense mutations were judged damaging by at least two of the three algorithms (Arg183Cys, Arg212Cys, Lys238Glu, and Pro392His). These four possibly damaging missense mutations were all present in patient and control subjects at similar frequencies. A fifth mutation, c.117_118insG(frameshift), was also judged to be likely detrimental as it leads to a series of 29 aberrant amino amino acids followed by a premature termination of the encoded protein after the 69^th^ amino acid. The c.117_118insG mutation was detected in 3 (0.27%) of 1098 population control subjects and was not identified in NTG patients. Overall, there was no skew in the frequency of the frameshift and four missense mutations judged to be potentially damaging between NTG patients and control cohorts (p = 0.31).

## Discussion

Recently, studies of glaucoma have identified disease-causing genes (*OPTN* and *TBK1*) that directly interact with each other in the same biological pathway. TBK1 encodes a kinase that phosphorylates OPTN and stimulates autophagy and mutations in TBK1 or OPTN appear to cause glaucoma via dysregulation of autophagy. Consequently, it was plausible to search for glaucoma-causing mutations in other autophagy genes. In addition to OPTN, TBK1 phosphorylates two other autophagy receptor proteins, SQSTM1 and calcium binding and coiled-coil domain 2 (CACOCO2). In this report we investigated the possible role of mutations in *SQSTM1* in NTG pathogenesis by testing a large cohort of NTG patients for glaucoma-causing mutations. We identified *SQSTM1* missense mutations in 11 (3.6%) of 308 NTG patients and in 2 (1.3%) of 157 normal controls, and in 32 (2.9%) of 1098 population controls.

Statistical analysis of the *SQSTM1* data identified no difference between the frequency of mutations in NTG patients and controls. Although the frequency of *SQSTM1* mutations in NTG patients was slightly higher in NTG patients, the difference was not statistically significant. These data suggest that *SQSTM1* mutations are not a common cause of NTG. However, it does remain possible that some *SQSTM1* mutations do cause NTG. For example, the Arg212Cys mutation was detected in one NTG patient and was absent from both control populations. Moreover, all three mutation analyses (SIFT, Blosum62, and PolyPhen-2) suggested that the Arg212Cys mutation may be pathogenic. Together these data raise the possibility that this specific mutation may cause disease. However, a much larger cohort would be required to detect disease-causing mutations with statistical significance. For example, a study of 5,000 NTG patients and 5,000 controls would have 76% power to detect a statistically significant difference between a 4% frequency of *SQSTM1* mutations in cases and a 3% frequency in controls. An alternative way to support a link between mutations in *SQSTM1* and NTG would be to compare inheritance of the detected mutations with the inheritance of glaucoma in family members. Unfortunately, this study utilized a cohort of unrelated NTG patients and additional family members are not available for study. Finally, the ultimate approach to investigate pathogenicity would be to generate a transgenic animals with rare *SQSTM1* variants to see if they develop glaucoma.

Mutations in the *SQSTM1* were previously associated with ALS. Interestingly, three of the same variants reported in ALS patients (Ala33Val, Val153Ile, and Pro392Leu)[33,34,40–45] were also detected in our population control cohort and are present at a similar rate in the ExAC database (Table 3). The presence of these variants in control subjects not known to have ALS might partially undermine their proposed role in the pathogenesis of this neurodegenerative disease.

There are potential limitations to our study. One method (Sanger Sequencing) was used to analyze the DNA of the NTG patients and the normal control subjects, while another method (whole exome sequencing) was used to analyze the DNA of the Iowa Population Control subjects. It is possible that these differences in methodology could bias to our results with different mutation detection efficiencies. However, we examined the whole exome sequencing data and found that there was an average of 88X coverage across the coding sequence of this gene in all subjects, which suggests that there was no bias against mutation discovery due to whole exome coverage. Also, both Sanger sequencing and whole exome sequencing detected non-synonymous mutations at similar frequencies (Table 4), suggesting that bias due to different sequencing methods is less likely. Our investigations of the *SQSTM1* gene were limited to coding sequences. It is possible that mutations involving non-coding, regulatory regions of the *SQSTM1* gene may be associated with NTG and went undetected by this report.

Future studies of larger cohorts with more extensive mutation detection strategies (i.e. assessing both coding and regulatory sequences) might have the power to identify rare glaucoma-causing *SQSTM1* variants. Alternatively, future association studies might also have the potential to identify common *SQSTM1* risk alleles for NTG. However, at present, the sum of our data suggests that coding sequence *SQSTM1* mutations are not associated with NTG.

## Supporting Information

S1 Table Variations detected in the SQSTM1 gene in control subjects by exome analysis. Column 1 (Variants detected in the SQSTM1 gene by the exome study) lists detected variants annotated relative to the hg19 reference genome. Column 2 (Variant location relative to SQSTM1 coding sequence) lists variants annotated relative to the transcript in HGVS standard nomenclature (http://varnomen.hgvs.org).
(XLSX)
